# Recommendations for a regional strategy for the prevention and control of emerging infectious diseases in the Americas.

**DOI:** 10.3201/eid0103.950312

**Published:** 1995

**Authors:** D B Epstein


CMV-antibody-negative or leukocyte-reduced cellular blood products. The guidelines also recommend that *Toxoplasma*-seropositive HIV-infected persons who have a CD4+ lymphocyte count <100 cells/µL received chemoprophylaxis against toxoplasmosis (such chemoprophylaxis is generally accomplished with anti-*Pneumocystis carinii* medication). Earlier recommendations for chemoprophylaxis against *Pneumocystis carinii* pneumonia and *Mycobacterium avium* complex disease have also been updated.

In addition to disease-specific recommendations, the guidelines include an overview article designed to prioritize the recommendations for health care providers. This article provides an approach to the initial and follow-up evaluations of the HIV-infected patient and also contains sections on HIV-infected pregnant women and HIV-exposed/infected children. The guidelines are followed by 15 background articles, which provide the information on which the recommendations were based and include research priorities generated by the development of the prevention recommendations.

The guidelines conclude with quality standards and implementation steps on the most standard-of-care recommendations, such as chemoprophylaxis against *Pneumocystis carinii* pneumonia. This final section provides a mechanism by which health care facilities can assess their degree of compliance with the recommendations, so that they can detect and correct compliance-related problems.

An abbreviated version of the USPHS/IDSA Guidelines will be published in CDC's *Morbidity and Mortality Weekly Report* in July.

**Jonathan E. Kaplan**
Centers for Disease Control and Prevention
Atlanta, Georgia, USA

**Henry Masur**
National Institutes of Health
Bethesda, Maryland, USA

**King K. Holmes**
University of Washington
Seattle, Washington, USA

### Recommendations for a Regional Strategy for the Prevention and Control of Emerging Infectious Diseases in the Americas

On June 14-15, 1995, a conference on "Combating Emerging Infectious diseases: Challenges for the Americas" was held at the Pan American Health Organization (PAHO) Headquarters in Washington, D.C. The meeting was designed to shape a regional strategy for preventing and controlling emerging infectious diseases that could pose serious threats to the peoples of the Americas.

Participants, convened by PAHO, included top officials and infectious disease experts from that organization as well as the World Health Organization, the U.S. Centers for Disease Control and Prevention, the Canadian Laboratory Center for Disease Control, the U.S. Department of Defense, and several Latin American and Caribbean countries.

This international group of experts noted that an increasing number of new, emerging, and reemerging infectious diseases have been identified in both developed and developing nations and that these diseases threaten to increase in the near future. They include human immunodeficiency virus/acquired immunodeficiency syndrome, which emerged in the l980s and now affects some 16 million people worldwide; and cholera, which returned to the Western Hemisphere for the first time this century in 1991 and has caused more than 1 million cases and 9,000 deaths in the Americas. PAHO estimates that it will take more than a decade and over $200 billion to control the current pandemic of this disease.

The experts concluded that both early warnings of, and rapid responses to, infectious disease threats are needed. The group made several major recommendations to PAHO and its member states to improve surveillance, research, and communications in developing countries. They also issued more detailed recommendations in the areas of antimicrobial resistance, outbreak control, and information and communication. In addition, a plan of action is forthcoming.

The group made the following recommendations for PAHO and its member countries:

### General Recommendations

- Develop and frequently update prioritized disease-specific guidelines for the prevention and control of diseases that are emerging or reemerging, both at the public health and individual levels. This should include biologic and behavioral change measures and will require groups of experts for each disease as well as communications experts. Diseases of interest include yellow fever, dengue, antimicrobial-resistant organisms (malaria, tuberculosis, and enteric diseases), measles, polio, cholera and other foodborne and waterborne diseases, viral hemorrhagic fevers, plague, rabies and other zoonoses, and trypanosomiasis and other vector-borne diseases.
- Identify points of contact in the field to receive and transmit information in countries. These contacts should include organizations and individuals outside the government.

- Develop plans to distribute accurate and timely information to the general public.
- Develop plans to improve and make more efficient two-way communication on reporting, control, and modification measures. This may require contracting information management specialists to identify and implement the most efficient means.
- Make efficient use of the press, including radio, television and newspapers, fliers, and other methods to educate the public and the medical community, with an eye toward social mobilization of communities to fight emerging diseases. This will require expertise in communications and support to the countries in developing information dissemination plans. Countries should define populations at greatest risk and focus the information and control measures in these populations.
- Define different approaches for educating the public and the medical community.
- Focus efforts on intersectorial action, including education of policy makers outside the health community.

### Antimicrobial Resistance

The expert group recommended that both PAHO and its member countries, where applicable, do the following:

- Seek ways to reduce availability of over-the-counter antimicrobial agents, including those used in veterinary medicine; this will require efforts beyond the health care community and involve education and dissemination of information to all sectors.
- Intensify assistance to the countries in developing rational drug policies.
- Monitor sensitivity to antibiotics in each country to allow for optimum antibiotic use for individual cases and to eliminate antibiotics with little therapeutic value. Employ mechanisms such as WHONET and PHLIS to centralize, analyze, and distribute antimicrobial sensitivity data.
- Develop and distribute specific recommendations to extend the useful life of antimicrobial drugs.
- Frequently revise the list of essential antimicrobials based on sensitivity data.
- Initiate educational campaigns on the cost-effectiveness of rational drug use in hospitals.
- Initiate collaboration with the pharmaceutical industry on rational drug use, standardized labels and warnings, and ethical marketing strategies.

### Outbreak Control

The expert group endorsed the leadership role of PAHO in developing and disseminating guidelines for outbreak evaluation and control and recommended that PAHO

- Make timely recommendations to coordinate response to outbreaks or threats, including issues related to travel advice, quarantine, and commerce.
- Develop policies and standard operating plans for response to outbreaks at the regional and country levels. Assist countries in developing national outbreak response plans and assist in training teams.
- Identify and list individuals and groups with disease-specific expertise, laboratories with disease-specific diagnostic capabilities, and products, including diagnostic reagents, drugs, and vaccines (both licensed and investigational products). Frequently update these lists.
- Establish a standard system for rapid procurement of vaccines, reagents, insecticides and antimicrobial drugs for prompt response to outbreaks.
- Establish information management and dissemination procedures for use during outbreaks, including accurate and frequent release of information to the press and public.
- Conduct formal evaluations of responses to each outbreak and use the lessons learned to improve responses to subsequent outbreaks.

### Information and Communication

The experts recommended communicating with high-level government officials and emphasizing to them the importance of a basic public health infrastructure—including improvements in water, sanitation, and social and economic conditions—in preventing diseases. The group suggested disseminating more information about public health implications of development (such as deforestation, dam construction, urbanization, and other measures) and seeking effective interaction with other sectors.

### Other Recommendations

PAHO should

- Create interagency task forces for emerging diseases at regional and country levels.
- Inform regional governments, other organizations, and the public about the emerging disease initiative and strive for the highest level of political support.

- Solicit and allocate specific resources to deal with the emerging diseases initiative, both at the regional and country levels. A portion of these funds should be immediately available when outbreaks are recognized.

For more information on these recommendations, the conference, or its plan of action, contact PAHO.

**Daniel B. Epstein**
Office of Information & Public Affairs
Pan American Health Organization
Washington, D.C., USA

### Emerging Infectious Diseases Laboratory Fellowship Program

A partnership has been established between the Association of State and Territorial Public Health Laboratory Directors and the Centers for Disease Control and Prevention (CDC) to develop and initiate an emerging infectious diseases laboratory fellowship program in January 1996. A goal of this fellowship program is to strengthen local, state, and federal public health infrastructures to support surveillance and implement prevention and control programs. The fellowship program will help recruit and train microbiologists for laboratories nationwide and provide opportunities for doctoral level scientists to conduct high-priority infectious disease research.

The emerging infectious diseases fellowship program will offer a 2-year laboratory research track for doctoral level scientists, with emphasis on applied research or development in infectious diseases and a 1-year advanced laboratory training track for bachelor's and master's level scientists, with emphasis on the practical application of emerging infectious diseases technologies, methods, and practices. Fellow training and research will take place at CDC and state and local public health laboratories.

For applications or additional information, contact

**Emerging Infectious Diseases Fellowship Program**
**Association of State and Territorial Public Health Laboratory Directors**
**1211 Connecticut Avenue, Suite 608**
**Washington, D.C. 20036**
**Phone: 202-822-5227, Fax: 202-887-5098**

### Tenth Annual ASTPHLD Conference on Human Retrovirus Testing

The Tenth Annual Conference on Human Retrovirus Testing, sponsored by the Association of State and Territorial Public Health Laboratory Directors (ASTPHLD), was held March 6 to 9, 1995, in Reno, Nevada. The conference, which was attended by more than 300 representatives of public and private sector laboratories as well as test kit manufacturers, emphasized three themes: new human immunodeficiency virus (HIV) variants, international issues, and HIV testing of newborns. The topics discussed included sequence data for type O isolates, the search for new HIV variants, zidovudine (AZT) resistance, decreased maternal-neonatal transmission due to AZT prophylaxis, results of the national anonymous survey of HIV prevalence in the United States, and the ethical concerns of perinatal screening.

An international perspective on HIV testing was brought to the conference by presentations that focused on India and Latin America. Results were given of a project, funded by a 12-month study grant from the World AIDS Foundation, to provide training on HIV testing to laboratories in India. Four Indian facilitators were trained in the United States; they provided translation and other assistance to eight ASTPHLD faculty, who gave workshops in four training centers in India. This training, which focused on enzyme immunoassay, linked trainees with staff from Indian reference centers and established training materials and trainers for future workshops to be conducted by Indian staff.

Laboratory aspects of HIV testing in Latin American and the Caribbean were also discussed by a member of the Pan American Health Organization (PAHO), who described the spectrum of HIV incidence rates and testing algorithms. PAHO is asking countries of the region to assess their algorithms in terms of sensitivity, specificity, and cost. PAHO aims to support national laboratories by providing guidelines and quality assurance. Proficiency testing, which is encouraged, will be provided by the Centers for Disease Control and Prevention.

ASTPHLD's 11th Annual Human Retrovirus Conference is set for March 6-8, 1996, in Orlando, Florida. Requests for additional information are available; FAX request to 202-887-5098.

**James L. Pearson**
Division of Consolidated Laboratory Services,
Commonwealth of Virginia, Richmond, Virginia, USA

